# Graphical Representation of Cell Count in Native Joint Aspirates

**DOI:** 10.3390/diagnostics16142222

**Published:** 2026-07-16

**Authors:** Marius Hoyka, Elke Weissbarth, Maike Keitel, Philipp Mayer, Irina Berger, Bernd Fink

**Affiliations:** 1 Department for Joint Replacement, Rheumatoid and General Orthopaedics, Orthopaedic Clinic Markgröningen, Kurt-Lindemann-Weg 10, 71706 Markgröningen, Germany; marius.hoyka@rkh-gesundheit.de; 2 Institute for Laboratory Medicine, Orthopaedic Clinic Markgröningen, Kurt-Lindemann-Weg 10, 71706 Markgröningen, Germanymaike.keitel@rkh-gesundheit.de (M.K.); 3 Department of Sports Medicine, Orthopaedic Clinic Markgröningen, Kurt-Lindemann-Weg 10, 71706 Markgröningen, Germany; philipp.mayer@rkh-gesundheit.de; 4 Institute of Pathology, Klinikum Kassel, Mönchebergstraße 41–43, 34125 Kassel, Germany; irina.berger@klinikum-kassel.de; 5 Orthopaedic Department, University Hospital Hamburg-Eppendorf, Martinistrasse 52, 20251 Hamburg, Germany

**Keywords:** synovial cell analysis, septic arthritis, crystal arthropathy, rheumatoid arthritis, haematoma, diagnosis

## Abstract

**Objectives**: The aim of the study was to describe the graphical synovial cell distribution as LMNE matrix patterns in native knee aspirates and to explore their association with septic arthritis, crystal arthropathy, rheumatoid arthritis and haemarthrosis. **Methods**: In total, 117 patients with knee joint effusions of various origins underwent aspiration of the knee joint and the aspirate analysed using the Yumizen H500 automated cell counter. Microscopy of the aspirate was performed at the same time in cases with a cluster in the so-called noise field or previously unknown cell distribution. **Results**: The graphical type differentiation of automated synovial cell count analyses of native joints allowed 9 different types to be recognised: Type I as particle type, type II as infection type, type III as combination type of infection and particles, type IV as indifferent type, type V as haematoma type and type VI as combination type of infection and haematoma, type VII as particle type with haematoma, type VIII in rheumatoid arthritis and type IX as mixed type combining infection and rheumatoid arthritis. Differentiation of LMNE types into infection types (types II, III, VI and IX) and non-infection types (types I, IV, V, VII and VII) resulted in a sensitivity of 100%, a specificity of 93.9%, an accuracy of 96.6%, a positive predictive value (PPV) of 92.7%, a negative predictive value (NPV) of 100%, a positive likelihood ratio (LR+) of 16.5 and a negative likelihood ratio (LR−) of 0. **Conclusions**: Graphical LMNE matrix analysis of native knee aspirates identified reproducible patterns associated with septic arthritis, crystal arthropathy, rheumatoid arthritis, and haemarthrosis, which may be helpful in distinguishing these clinical entities, suggesting that graphical cell count interpretation may provide useful adjunctive information to the standard diagnostic work-up. The study is a preliminary descriptive study and requires validation using a larger number of patients.

## 1. Introduction

The synovial fluid cell count represents a pivotal diagnostic tool for rapidly determining the etiology of joint effusions. This is particularly essential for the diagnosis of septic arthritis, which is a severe condition that can cause rapid destruction of the articular cartilage as well as life-threatening systemic consequences [1,2,3]. Pathogen identification through microbiological cultivation often requires several days; thus, initiating treatment typically cannot be delayed while awaiting these outcomes [3].

In the absence of leukopenia, many authors advocate a cut-off value of 50,000 cells/μL for septic arthritis in native joints [3,4]. However, this cut-off value is not always appropriate; for instance, Eren et al. [5] identified pathogens in the aspirate of 15.9% of cases (26 of 192 patients) with a cell count below 50,000 cells/μL, while McGillicuddy et al. [6] reported this in 39% of cases. Furthermore, Ruzbarsky et al. [7] reported a “gray zone” of 25,000 to 75,000 cells/μL in the synovial aspirate, within which the diagnosis of septic arthritis remains unclear. In the postoperative setting, Voss et al. [8] defined a cut-off value of 20,000 cells/μL in the synovial aspirate for septic arthritis after arthroscopic interventions.

Other arthropathies can also exhibit elevated cell counts within this “gray zone” in the absence of septic arthritis. For example, crystal arthropathies or inflammatory rheumatic diseases, such as rheumatoid arthritis, can demonstrate increased cell counts in the synovial fluid [9,10,11]. Therefore, differentiating between acute aseptic arthritis—such as an acute gouty flare in crystal arthropathy or an inflammatory flare-up in rheumatoid arthritis—and septic arthritis based solely on cell count determination is often difficult, as both can present with similarly high cell counts in the aspirate and comparable local clinical symptoms [10,12,13]. In addition, both conditions can also occur simultaneously [12,14].

Graphical cell count analysis of synovial aspirates is a novel, helpful method for differentiating leukocytes in the aspirate from particles (such as wear debris from implanted endoprostheses), which are otherwise counted during automated cell count analysis [15,16,17]. In hip and knee arthroplasty, this significantly increased the diagnostic value of cell count determination and reduced misdiagnoses of periprosthetic infections in the presence of wear particles [15,16,17]. Seven distinct types of graphical patterns have been identified for indwelling prostheses: type I (abrasion type), type II (infection type), type III (combination of infection and abrasion), type IV (indifference type), type V (haematoma type), type VI (combination infection and hematoma), and type VII (particle type with hematoma) [15,16,17,18].

The present study aims to investigate whether distinct types can also be identified via graphical cell count analysis of synovial aspirates from native joints (without an endoprosthesis). Furthermore, we aimed to test the hypothesis that the seven types described in joints with endoprostheses can also be found in native joints, whereby crystals, for example, generate these patterns in native joints instead of the wear debris observed in types I, III, and VII.

## 2. Materials and Methods

Between March 2022 and September 2025, 117 patients with an effusion of a native knee joint (without a knee prosthesis) underwent arthrocentesis. Concurrently, 28 patients had rheumatoid arthritis and 10 patients had crystal arthropathy. In 23 patients, the aspirate was obtained postoperatively following anterior cruciate ligament reconstruction or osteosynthesis of the knee. The cohort comprised 54 women and 63 men with an average age of 63.82 ± 13.76 years (range: 24.4–90.5 years).

The aspirate was analysed using the Yumizen H500 automated cell counter (HORIBA ABX SAS, Grabels-Montpellier, France). The Yumizen H500 is an automated laboratory diagnostic device for analysis, counting and differentiation of leukocytes in blood and other body fluids. By using the so-called 5-DIFF processing mode, a total of 26 laboratory parameters including the 5 cell types lymphocytes, monocytes, eosinophils, neutrophils and basophils as well as large, immature cells and atypical lymphocytes are available to the investigator. Additionally, these cell types are arranged in a cell distribution chart, the so-called LMNE-Matrix, based on the cell volume difference defined by impedance measurement (X-axis) and the differentiation of the light absorption in flow cytometry (Y-axis). This allows the leukocyte populations to be graphically assigned and, consequently, differentiated, whereas impurities (for example abrasion or wear particles in joints with endoprostheses) are localized. In patients in whom a cluster could be recognized in the so-called noise field of the LMNE matrix or in cases of a previously unknown cell distribution, the aspirate was additionally examined by microscopy. The evaluations and assignment of the individual matrices to the different types of image were carried out twice by 2 examiners (MH and BF) independently of one another. It showed a high reliability, with an intrarater intraclass correlation coefficient of 0.99 and of 0.98 between raters, respectively, as well as a Cohen’s kappa of 0.97. In addition, the serological parameters CRP and leucocytes as well as uric acid in the blood were recorded.

The diagnosis of septic arthritis was defined as either a positive bacterial culture or the joint presence of a high synovial cell count of >20,000/μL and PMN > 70% and an elevated CRP [8]. Crystal arthropathies such as gout or chondrocalcinosis were either known anamnestically or were defined when crystals were detected in LMNE type I with clusters in the noise field and by microscopic study of the aspirate [2]. In patients with rheumatoid arthritis, the diagnosis was already known in advance. A haemarthrosis was defined by the presence of blood and a peak in the additional erythrocyte curve of the cell count analysis in the Yumizen H500.

SPSS for Windows (version 22; IBM Corp.; Armonk, NY, USA) was used for statistical analysis. All values are either given as mean and standard deviation. For statistical evaluation of parametric data, a Student t-test and for nonparametric data, a Mann–Whitney U-test was used. All reported *p*-values are two-tailed, with an alpha level <0.05 considered as significant. All subjects gave their informed consent for inclusion before they participated in the study.

## 3. Results

### 3.1. LMNE Types

All 7 LMNE types described so far were also seen in the native aspirates. The classification of the aspirates into the different LMNE types and their parameters are listed in Table 1.

**Figure 1:**
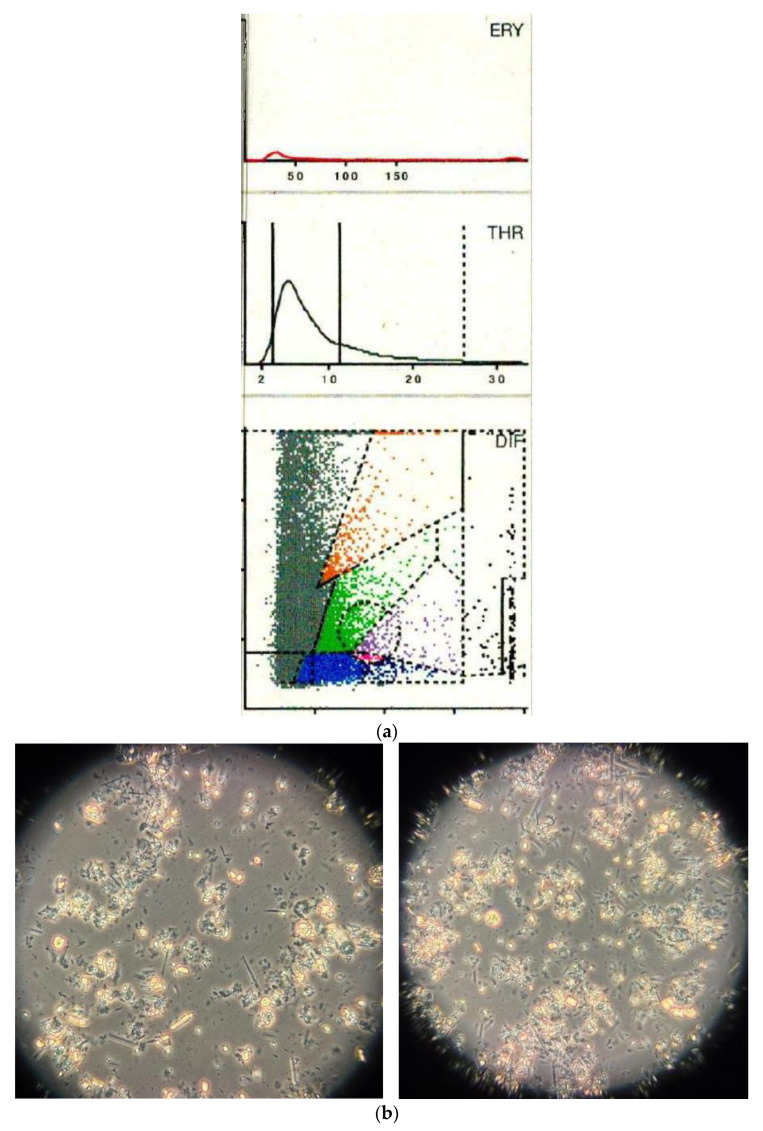
(**a**) LMNE matrix of type I (particle type) of a native knee aspirate from a patient with large clusters in the noise field (gray clusters on the far left). The cell count was 82,300/μL with a percentage of polymorphonuclear neutrophil granulocytes (PMN%) of 26.2%, the serum CRP value was 53.5 mg/L and the uric acid value was 10.8 mg/dL. The patient was known to have crystal arthropathy (gout). (**b**) Crystal particles in the microscopic examination of the aspirate (magnification: ×1000).

**Figure 2:**
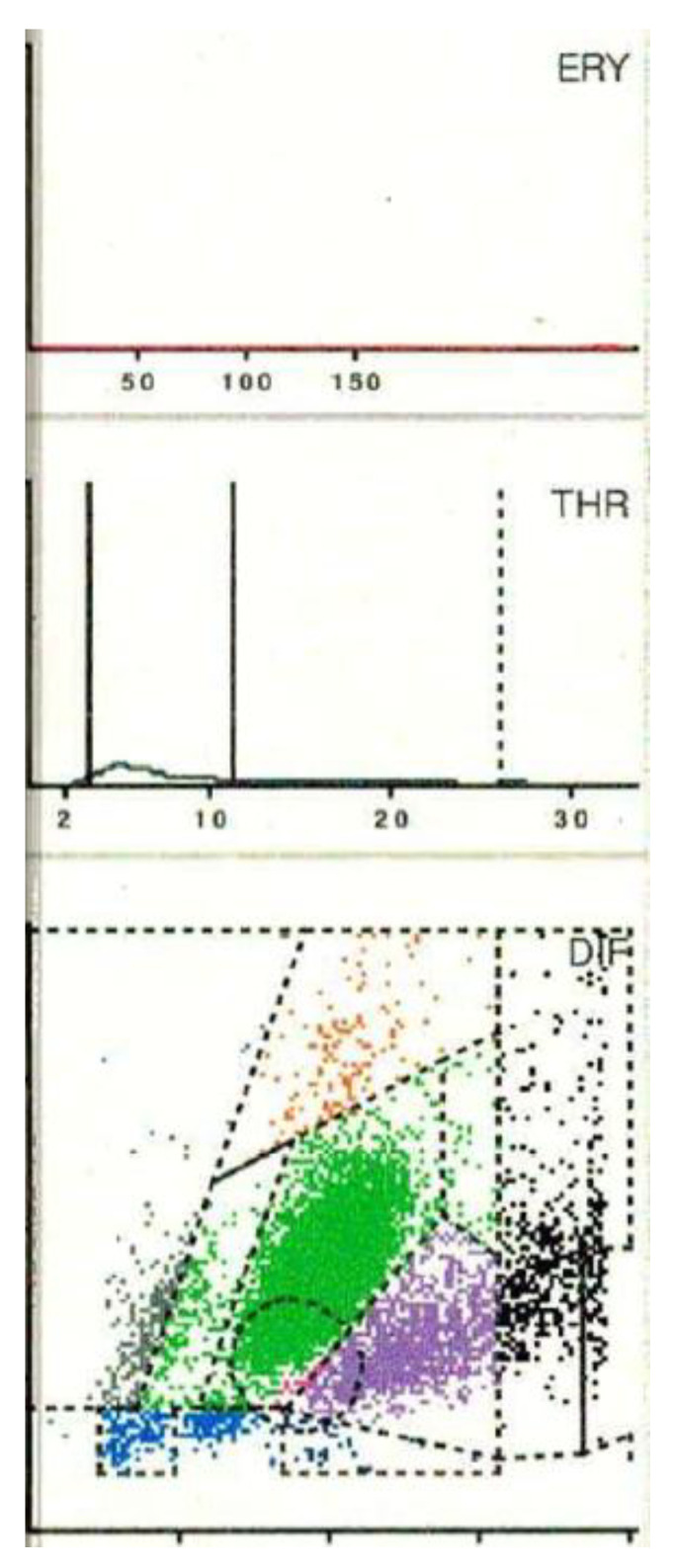
LMNE matrix type II (infection type) of a knee aspirate from a case with septic arthritis after ACL reconstruction with a significant increase in neutrophil granulocytes (green clusters in the middle). The synovial cell count was 24,210 cells/μL with a proportion of polymorphonuclear neutrophil granulocytes of 91.2%. The serum CRP was 93.4 mg/L and the leucocyte count was 8610/μL. Microbiological cultivation revealed the presence of *Staphylococcus aureus*.

The particle type (type I) was observed in two cases, in which a cluster was visible in the so-called noise field (Figure 1a). In both cases, a history of crystal arthropathy was known, and crystals were detected via light microscopy of the aspirates (Figure 1b). Type II (infection type) was identified 40 times, showing a distinct cluster in the polymorphonuclear leukocyte field (Figure 2). Among these, two patients had rheumatoid arthritis and 6 patients had crystal arthropathy. In 38 of these 40 patients, the findings were consistent with septic arthritis.

Microbiological detection was successful in 28 cases (14 times *Staphylococcus aureus*, five times *Cutibacterium acnes*, three times *Streptococcus dysgalactiae*, two times *Escherichia coli*, two times *Finegoldia magna*, once *Staphylococcus epidermidis*, and once *Streptococcus pneumoniae*) (Table 2). No microorganisms were detected in 10 patients. In all of these cases, the patients had received prior antibiotic treatment elsewhere. Notably, 32 of these patients exhibited a cell count in the aspirate below the cut-off value of 50,000/μL (Table 3).

**Figure 3:**
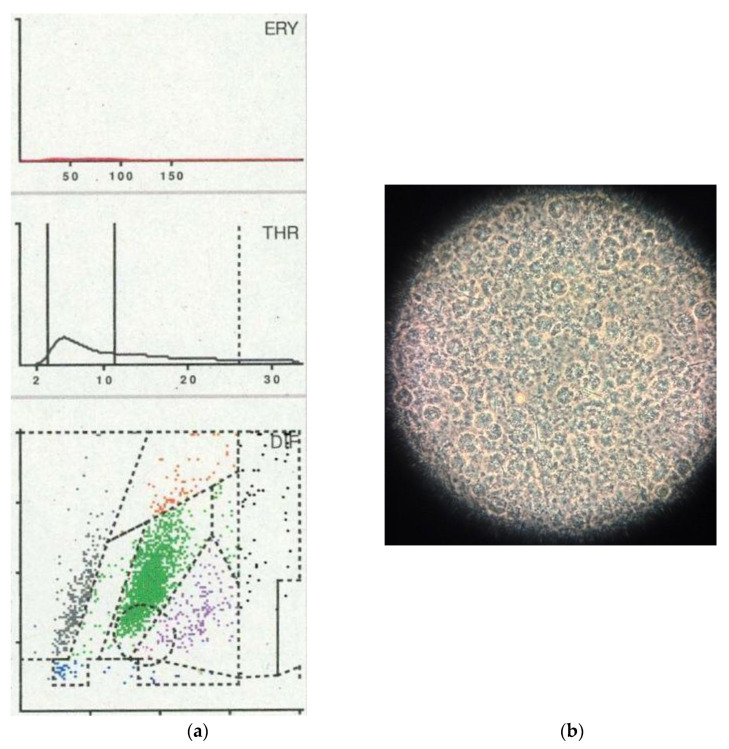
(**a**) LMNE matrix of mixed type III (particles and infection) with a cluster both in the noise field (gray clusters on the far left) and in the neutrophil granulocyte area (green clusters in the middle) in a patient with septic arthritis following an outpatient knee joint aspiration. A later histopathological study revealed evidence of concomitant chondrocalcinosis (**b**). The synovial cell count was 26,100 cells/μL with 88.7% PMN. The serum CRP was 117 mg/L. Microbiological cultivation revealed *Staphylococcus warneri* as the causal pathogen. (**b**) Crystal particles in the microscopic examination of the aspirate (magnification: ×1000).

**Figure 4:**
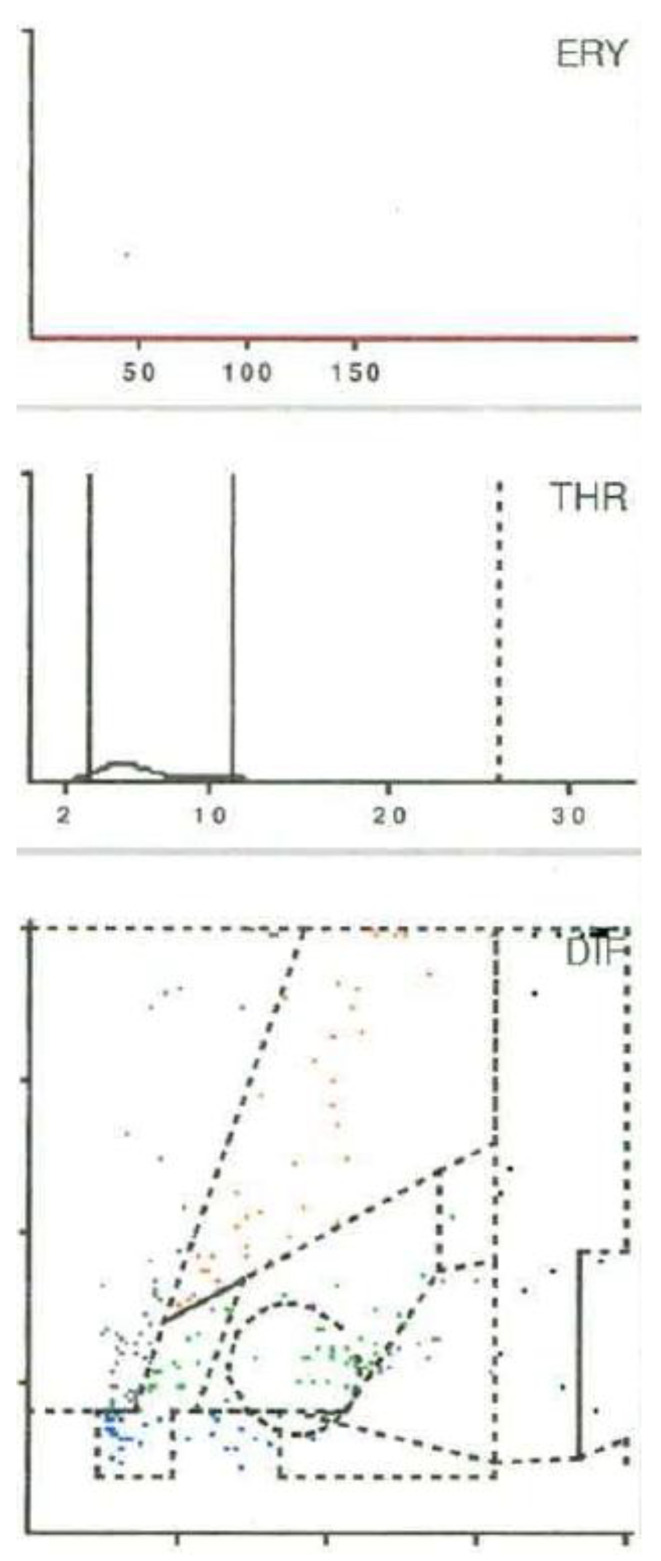
LMNE matrix of indifference type IV from a patient with a knee joint effusion without a clear distribution pattern or accumulation of cells and particles. The synovial cell count was 400 cells/μL and the proportion of polymorphonuclear neutrophil granulocytes was 31.1%. The serum CRP was 1.0 mg/L and the leucocyte count was 12,070 cells/μL. Microbiological incubation yielded no evidence of pathogens.

**Figure 5:**
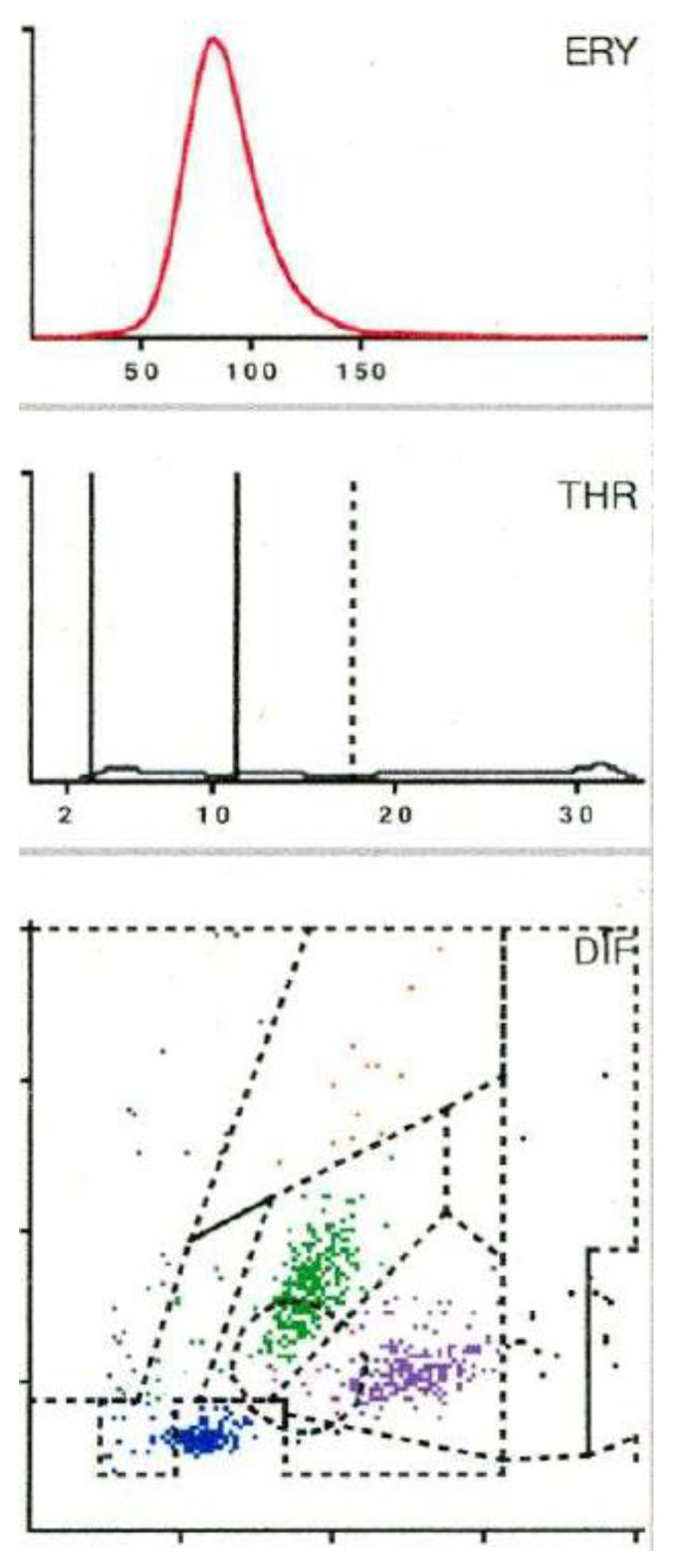
LMNE matrix of type V in a patient presenting with pronounced haemarthrosis following ACL plastic surgery. The additional erythrocyte curve provided in the punctate analysis shows a high amplitude. The LMNE matrix also showed a discrete increase in lymphocytes (blue clusters at the bottom left) and neutrophils (green clusters in the middle) with a cell count of 1060 cells/μL. The serum CRP was unremarkable at 4.9 mg/L and leucocytes at 4950/μL.

**Figure 6:**
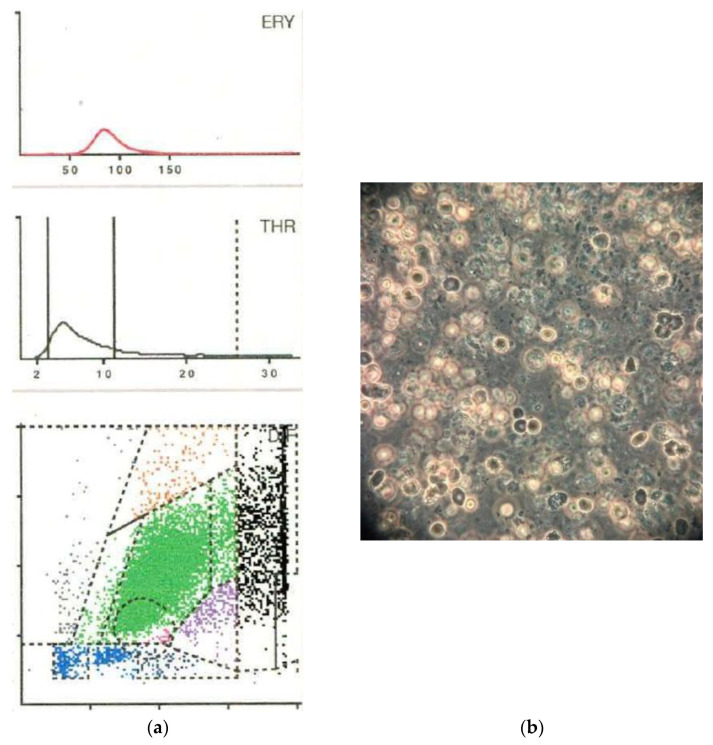
(**a**) LMNE matrix of mixed type VI consisting of haematoma and infection from a patient with infected haemarthrosis following knee joint arthroscopy. In addition to a spike in the erythrocyte curve, there was a pronounced cluster in the neutrophil granulocyte area (green clusters in the middle). The synovial cell count was 27,980 cells/μL with a percentage of polymorphonuclear neutrophil granulocytes of 88.3%. The biopsies obtained during the study showed an increased cell count with >50 NG/HPF. *Cutibacterium acnes* was detected in microbiological analysis. The laboratory parameters showed a serum CRP of 99.4 mg/L. (**b**) Microscopic analysis of the aspirate reveals large numbers of erythrocytes (magnification: ×1000).

**Figure 7:**
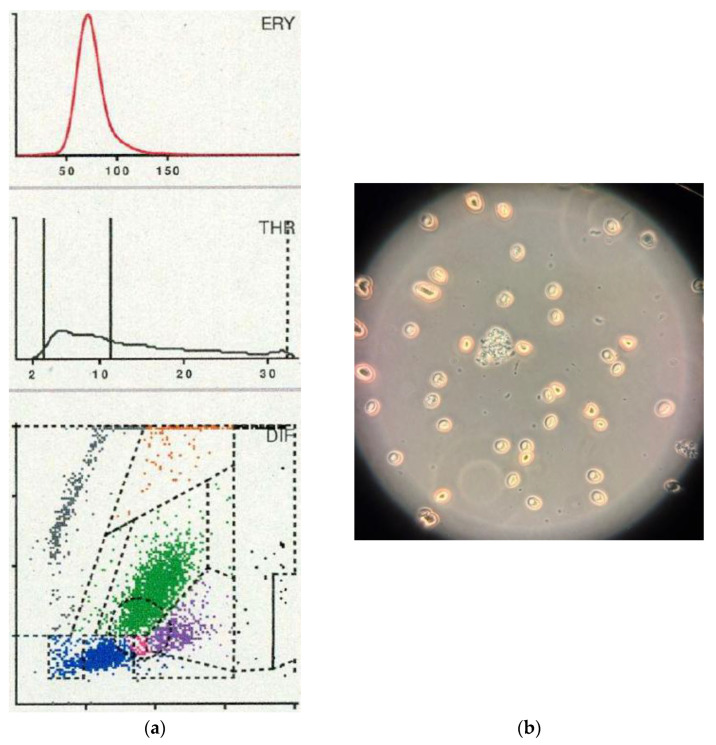
(**a**) LMNE matrix of mixed type (type VII) consisting of haematoma and particles from a patient after multiple osteosynthetic procedures on the knee joint. Particles in the form of clusters can be seen in the noise field (gray clusters on the far left). The post-surgery haemarthrosis is represented by a significant increase in the erythrocyte curve and additional clusters in the lymphocyte and neutrophil areas. The measured cell count was 10,300 cells/μL with 53.4% PMN. The serum CRP was 13 mg/L and the leucocyte count was 8170/μL. No pathogens were detected in microbiological culture. (**b**) Microscopy of the aspirate showing erythrocytes and particles (magnification: ×1000).

Type III was identified in two cases, presenting with one cluster in the noise field and another in the field for polymorphonuclear leukocytes as a mixed type (Figure 3a). Both patients had a known history of crystal arthropathy, which was confirmed microscopically (Figure 3b). Furthermore, pathogen isolation was successful in both cases (*Staphylococcus aureus* and *Staphylococcus warneri*). The so-called indifference type (type IV) was diagnosed in 36 cases, in which no clusters were observed (Figure 4). Three patients in this group had a known history of rheumatoid arthritis. The haematoma type (type V) was observed in eight cases. In addition to small clusters in various leukocyte fields (neutrophil granulocytes, monocytes, and lymphocytes), a peak in the erythrocyte curve was present (Figure 5). None of these patients had a known concomitant disease related to any form of arthritis. Another patient presented with a mixed type combining infection and haematoma (type VI), which was characterized by a large cluster in the field of polymorphonuclear leukocytes indicating infection, and a peak in the erythrocyte field (Figure 6a). Microscopic examination revealed a significant accumulation of erythrocytes (Figure 6b). The mixed type combining haematoma and particle presence (type VII) was found six times. A cluster in the noise field, smaller clusters in the leukocyte fields, and a peak in the erythrocyte curve were observed in these cases (Figure 7a). The combination of erythrocytes and particles was confirmed during microscopic examination (Figure 7b). Similarly, no patient in this group had an arthritic concomitant diagnosis.

**Figure 8:**
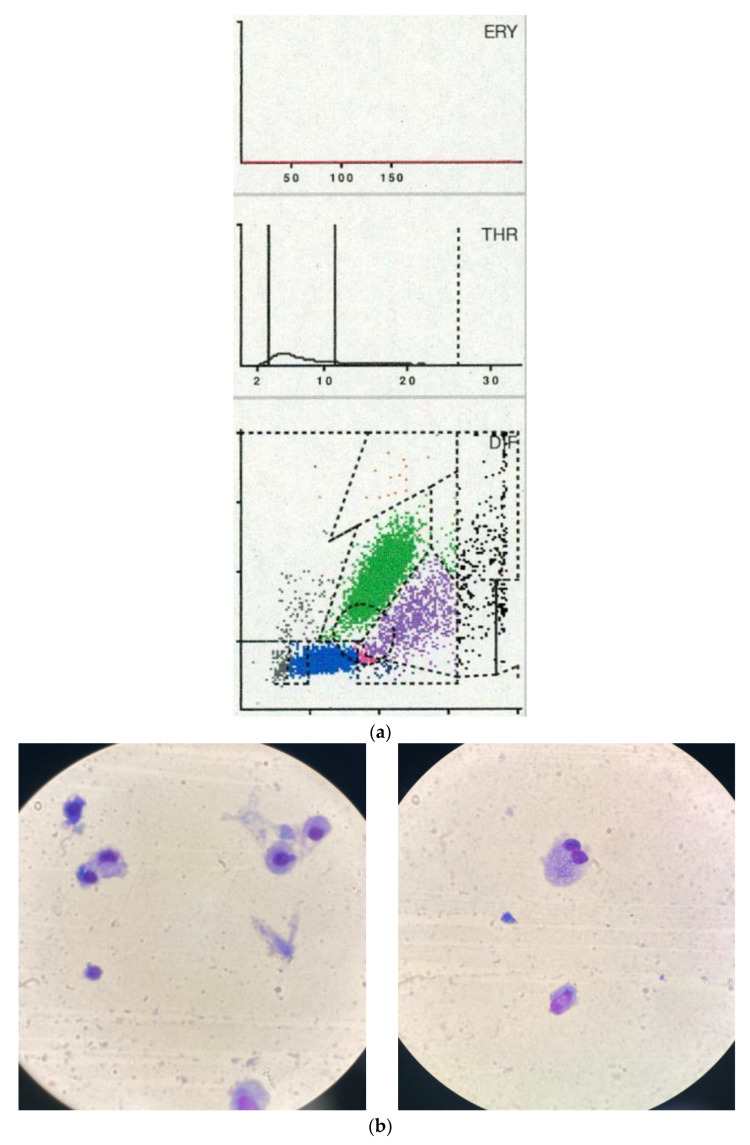
(**a**) LMNE matrix of the rheumatoid type (type VIII) from a patient with aspiration of the knee joint during an acute rheumatic attack. In addition to clusters in the field of neutrophils (green clusters), lymphocytes (blue clusters) and monocytes (purple clusters), numerous large cells (black clusters) are also shown on the far right. The proportion of large immature cells was 3.1%. The measured cell count was 19,300 cells/μL with 63.1% PMN. The serum CRP was 12.2 mg/L and the leucocyte count was 6250/μL. (**b**) Microscopy of the aspirate showing macrophages (magnification: ×1000, staining: May-Grünwald).

A total of 28 patients were known to have rheumatoid arthritis, with 12 of these cases concurrently diagnosed with septic arthritis. In 12 of the remaining 16 cases, where there was no evidence of septic arthritis, a cell distribution was observed that resembled the infection type II; however, it could be distinguished by the presence of additional clusters in the lymphocyte and monocyte fields, alongside the cluster in the polymorphonuclear leukocyte field. In 10 of these 12 cases of this previously undescribed rheumatoid type (type VIII), an additional accumulation was noted in the area of large cells (on the right side of the matrix) (Figure 8a). This was also reflected in the increased percentage of large immature cells (LIMC), with a mean value of 13.06 ± 22.29% (reference: 0.0–2.0%). Microscopy revealed the presence of macrophages in the aspirates of these patients (Figure 8b).

**Figure 9:**
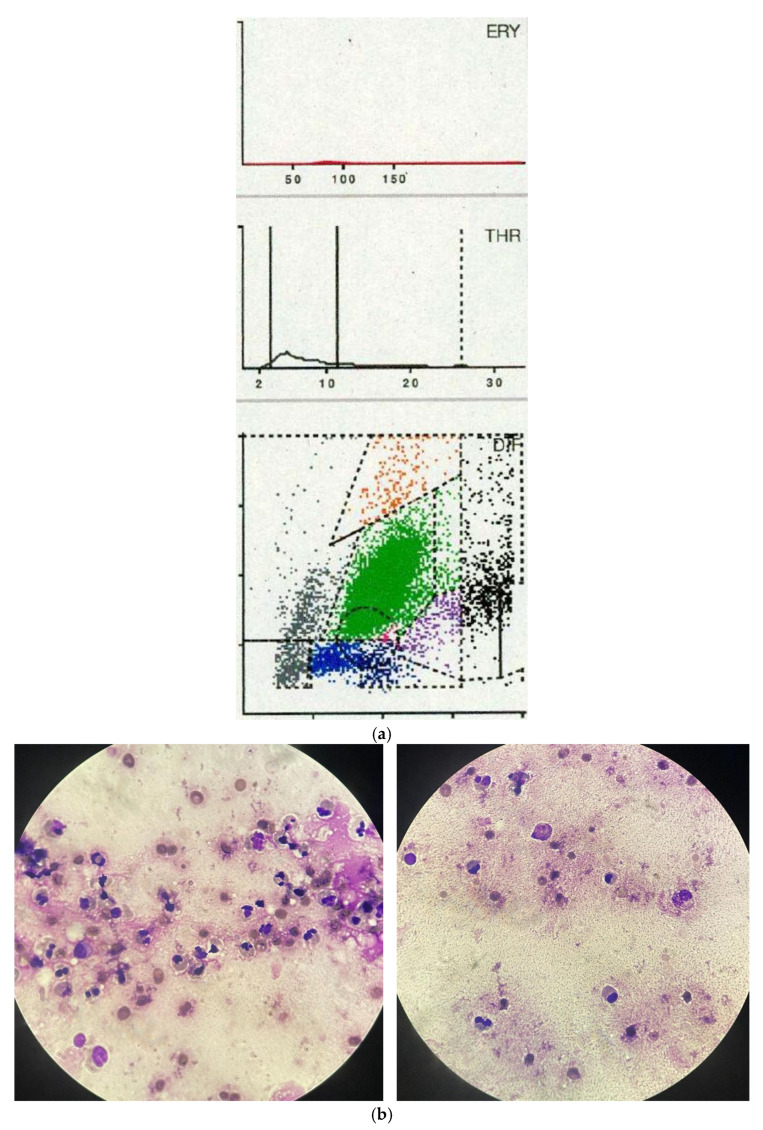
(**a**) LMNE matrix of mixed type (type IX) from a patient with known rheumatoid arthritis who presented with suspected haematogenous dissemination in the presence of spondylodiscitis. In addition to a marked increase in neutrophil granulocytes (green clusters), large cells (black clusters) are also seen on the far right of the matrix. The sample analysis revealed a synovial cell count of 60,480/μL and 91.5% PMN. The serum CRP was 158 mg/L. Microbiological cultivation revealed *Staphylococcus aureus* as the causal pathogen. (**b**) Microscopy of the aspirate showing leukocytes and macrophages (magnification: ×1000, staining: May-Grünwald).

In a further 10 patients with known rheumatoid arthritis, more pronounced clusters were identified in the field of polymorphonuclear leukocytes as well as lymphocytes and monocytes, in addition to the increased accumulation in the large cell area. This defined another mixed type (type IX) between septic arthritis and rheumatoid arthritis (Figure 9a). Microscopic examination revealed a combination of leukocytes and macrophages (Figure 9b). In seven cases, microbiological culture of the aspirate resulted in the detection of *Staphylococcus aureus* (four times), *Streptococcus dysgalactiae* (once), *Staphylococcus epidermidis* (once), and *Escherichia coli* (once).

Table 4 provides an overview of the various types of LMNE, along with their graphical features, typical diagnosis and the required confirmatory tests.

The cell count was significantly higher in group IX with 39,706.4 ± 23,995.85/μL than in the group of type VIII with 12,956.67 ± 6641.03/μL (*p* = 0.022). Furthermore, the number of polymorphonuclear leukocytes tended to be higher in group IX (79.12 ± 11.18%) than in group VIII (67.5 ± 8.11%, *p* = 0.057) (Table 5).

### 3.2. Clinical Diagnoses

Septic arthritis was present in 51 cases (Table 5). Synovial fluid analysis in this group demonstrated an average cell count of 31,162.82 ± 20,855.45/μL (range = 9200–73,400/μL) and an average proportion of polymorphonuclear leukocytes of 81.89 ± 9.75% (57.5–92.9%). The cell count was below 50,000/μL 39 times (Table 3). Pathogen detection was successful in 38 cases (Table 2). The remaining 13 cases had received prior antibiotic treatment, and no pathogens could be isolated in these cases. The serological parameters CRP (149.76 ± 109.52 mg/L; 39–427 mg/L) and leukocyte count (12,860 ± 3670/μL; range = 6940–19,910/μL) were consistently and significantly elevated in the septic arthritis group.

Crystal arthropathy was identified in a total of ten patients, with eight of these cases also diagnosed with septic arthritis. In the remaining two cases, concomitant with an increased cell count (82,128 ± 4635/μL; range = 77,493–86,763/μL), an increased percentage of LIMC (3.5 ± 0.4%, range = 3.1–3.9%) and elevated uric acid levels (10.5 ± 1.45 mg/dL; range = 9.1–12 mg/dL) were also observed (Table 5).

The 16 cases of rheumatoid arthritis without septic arthritis demonstrated an average synovial cell count of 10,096.25 ± 7953.18/μL, range = 520–20,900/μL). The proportion of large immature cells was also elevated (12.47 ± 20.69%; range = 1.7–62.9%) (Table 5).

In a total of 15 cases (including one case with septic arthritis), a haematoma was detected via the additional erythrocyte curve in the cell count analysis (eight times type V, once type VI, and six times type VII), with the cell count remaining below 50,000/μL in all aspirates. No previous arthritic disease was documented. After excluding the single case of mixed infection and haematoma (type VI), the cell count in the 14 patients with a pure haematoma was slightly elevated at 4802.85 ± 3684.1/μL (range = 650–10,300/μL) with an unremarkable proportion of polymorphonuclear neutrophil granulocytes (50.17 ± 11.29%, range = 34.5–65.9%) (Table 5).

A reactive effusion was diagnosed in the remaining 34 cases. The distribution of the LMNE matrix analysis revealed 32 cases of type IV and two cases of type II. None of these patients suffered from crystal arthropathy or rheumatoid arthritis. Both the synovial cell count with an average value of 2491.23 ± 7743.07/μL (range = 145–33,410/μL) and the serum CRP 11.18 ± 10.55 mg/L (range = 1–33.1 mg/L) were slightly elevated (Table 5).

### 3.3. Statistical Analysis

To evaluate the diagnostic value of the graphical LMNE matrix in differentiating septic from aseptic arthritis, synovial fluid analysis patterns from a total of 117 samples were evaluated (Table 6). Classifying infectious LMNE types (types II, III, VI and IX) as a positive test result demonstrated excellent discriminatory power. Among the patients displaying infectious LMNE types, 51 were confirmed to have septic arthritis (right positive), while 4 cases were clinically classified as aseptic (false positive). Conversely, non-infectious LMNE types (types I, IV, V, VII and VIII) were associated without septic cases (false-negative) and 62 correctly identified aseptic cases (right-negative). This distribution yielded a sensitivity of 100%, a specificity of 93.9% and an accuracy of 96.6%. The positive predictive value (PPV) was 92.7%, and the negative predictive value (NPV) reached 100%. Analysis of probability ratios revealed a positive likelihood ratio (LR+) of 16.5 and a negative likelihood ratio (LR−) of 0.

## 4. Discussion

The seven types previously described for aspirates from joints with prosthetic implants were also detectable in native joints, although instead of abrasion particles (such as polyethylene or metal from endoprostheses), crystals were identified in the aspirates of the native joints in this study.

The joints with detected infections did not consistently show a cell count above the specified cut-off value of 50,000 cells/μL in the synovial aspirate; in 39 of the 51 septic arthritis cases (76.47%), the cell count was below this cut-off value. This is a well-known phenomenon in the published literature [2,5,6,7]. George et al. [19] observed an average cell count of 13,561 cells/μL in aspirates from septic arthritis in adults. The cause may be antibiotic therapy administered prior to aspiration, as this is known to reduce the cell count in the aspirate [20,21]. This was the case in 13 patients in our study. In all of these septic arthritis cases, no pathogens were detected, which may also be related to the administered antibiotic therapy. Other authors also reported a lack of bacterial detection in septic arthritis of between 7% and 34% [2,22,23].

Another explanation for the reduced cell count in the aspirate may be an impaired immune defence due to immunomodulating diseases or disease-modifying antirheumatic drugs (DMARDs), as these therapies can reduce the number of cells in the aspirate [24,25]. This may explain the lower counts observed in patients with rheumatoid arthritis who are receiving these disease-modifying drugs.

In these cases, the additional information provided by the graphical type classification seems to be helpful for diagnosing septic arthritis, even below the cut-off value, by supporting the clinical assessment and initiating prompt treatment. The statistical analysis underscores the clinical value of the LMNE matrix. However, this graphical analysis should not be viewed as a standalone tool or a replacement for standard microbiological cultures and clinical work-up, but rather as a rapid screening aid that prompts earlier clinical suspicion. 

Moreover, in our opinion, the graphical representation of synovial cell count analysis is also helpful in distinguishing septic arthritis from aseptic arthritis such as crystal arthropathies or rheumatoid arthritis as well as haematomas and appears promising as an adjunctive diagnostic tool joint with unclear joint efusions.

The crystals were detected in the so-called noise field of the LMNE matrix. Previous work on synovial aspirates in joints with endoprostheses has already demonstrated the deposition of wear particles (type I) in this area [15,16,17]. In the present study, however, crystals rather than abraded particles accounted for the display in this field. This phenomenon has not previously been described in native joints. The significantly increased synovial cell count in the crystal arthropathy group is thus due to the false-positive counting of crystal particles, a phenomenon already discussed in the literature [9]. In our study, the cell count in the two patients with crystal arthropathy without infection was even higher than in the septic arthritis cases. Thus, the graphic type representation seems to be of particular value in septic arthritis with cell counts in the aspirate below the cut-off value of 50,000 cells/μL for differentiating it from aseptic arthritides, such as crystal arthropathies.

This graphical type differentiation also seems to be helpful for the differentiation of septic arthritis with lower cell counts and aseptic arthritis from haematomas. The so-called erythrocyte field is of particular importance here, as a peak in this field makes it easy to recognize the blood contamination of the aspirate. This had already been described in previous studies of synovial aspirates from joints with endoprostheses [16,26].

It was difficult to differentiate the LMNE matrix in patients with rheumatoid arthritis (type VIII) from infection type II, as both types had a cluster in the field of polymorphonuclear leukocytes. In rheumatoid type VIII, there was also an accumulation in the lymphocyte and monocyte field, which is much less pronounced in the pure infection type and only small accumulations of lymphocytes and monocytes are present [17]. In rheumatoid joints, there may be an increased number of leukocytes in the aspirate, up to 50,000 cells/μL, and accumulations of lymphocytes and monocytes are also frequently seen in aspirates from rheumatoid joints [9,10,11,27]. The accumulation of cells in the right matrix area, which we observed in 10 of 16 patients with rheumatoid arthritis without infection, may also be helpful in identifying a rheumatoid joint. This accumulation of large cells in the right matrix area could be explained by macrophages in the aspirate of patients with rheumatoid arthritis, as these have a larger cell volume and could be counted as large immature cells (LIMC) in the cell count analysis because of their cell size, although the macrophages are mature cells. In all cases in our study with these LMNE types VIII or IX, macrophages were seen by microscopy. Macrophages in the aspirate of patients with rheumatoid arthritis are a well-known observation [28,29,30].

However, it is no longer possible to differentiate between type VIII and type IX, the rheumatoid joint with infection, by graphical representation alone, as the two types differ only in the extent of the accumulation of cells in the field of polymorphonuclear leukocytes and thus also in the level of the total cell count. Here, the percentage of polymorphonuclear leukocytes is decisive for differentiation, as it was significantly higher in group IX than in group VIII.

Manual counting could be considered as an alternative to the common method of automated cell counting of synovial aspirates. However, this is significantly more time-consuming than automated counting [31]. In addition, manual counting also exhibits inaccuracies. In turn, manual counting has been shown to result in an inter-observer variance of more than 20% reducing the accuracy of manual counting of WBC in synovial fluid compared to automated counting [32]. The regular microscopy of synovial fluids also has the disadvantage of additional complexity.

Other parameters such as synovial lactate measurement are also suitable for differentiating between septic and aseptic arthritis, e.g., gouty arthritis [13]. However, synovial lactate measurement is not routinely analysed in the automated cell count and therefore represents an extra study. In contrast, the graphical cell count analysis with the LMNE matrix is automatically performed in the analyser used here.

This study has limitations. First of all, this is a preliminary study, and it is the first description of this graphical type differentiation of synovial cell counts of native joints. The number of cases was too low for a statistical analysis of all subtypes. Consequently, the statistical analysis was carried out for all infection types and all non-infection types combined. Thus, in subsequent studies with a large number of patients, this type classification for native joints as well as statistical analysis for all subtypes must be verified. Moreover, this study was limited by its single-center design, meaning our findings may have been influenced by local institutional factors. These include the logistical workflows and turnaround times of our in-house laboratory, the specific demographics of the local patient cohort, and the individual clinical thresholds used by attending physicians to determine the indication for joint aspiration. Consequently, future external validation within a multi-center study design is warranted to confirm the generalizability of our data. Additionally, the analyzed patient cohort was restricted exclusively to knee joints. Because both the biochemical composition of the synovial fluid and the pathophysiological dynamics of inflammatory processes can differ significantly between the knee and other joints, our results cannot be directly extrapolated to other anatomical sites. Therefore, further investigations are required to systematically evaluate the applicability of this method to other joint types. Another important methodological limitation is the potential for incorporation bias within our reference standard for septic arthritis. Because both the synovial cell count and the PMN percentage were generated by the same automated system being evaluated for the LMNE pattern, these parameters are inherently interdependent. However, the automatic cell counter used in this study does not allow a separate analysis of the LMNE typing from the measurement of the cell count and the percentage of polymorphonuclear leukocytes. Future studies should therefore aim to evaluate the index test independently from the reference standard criteria to eliminate this bias. Furthermore, a microscopic study as well as histological analysis was not carried out on all aspirates at the same time, but only as an exploratory analysis on types with suspected simultaneous presence of crystals or previously unknown cell distributions. In our opinion, this was only necessary in such cases in order to see what was also counted during the automated counting. Furthermore, graphical type differentiation is a qualitative analysis and involves a certain degree of subjectivity. However, in the previous studies on joints with endoprostheses, this examination method showed a very high diagnostic value and the inter- and intraobserver correlation in this study was high [15,16,17,18].

In summary, graphical LMNE matrix analysis of native knee aspirates identified reproducible patterns associated with septic arthritis, crystal arthropathy, rheumatoid arthritis, and haemarthrosis. These findings suggest that graphical cell count interpretation may provide useful adjunctive information, particularly when conventional cell count thresholds are inconclusive. Larger prospective studies using standardized microscopy and independent diagnostic reference standards are required to validate the diagnostic accuracy of the graphical LMNE matrix analysis.

## Figures and Tables

**Table 1:** Parameters for the different LMNE-types (mean ± standard deviation; minimum–maximum).

	N	Cell Count (/μL)	% PMN	% LIMC	CRP (mg/L)	Leukocytes (10^3^/μL)	Uric Acid (mg/dL)
Type I	2	82,128 ± 4635 (77,493–86,763)	26.2 ± 2.7 (23.5–28.9)	3.5 ± 0.4 (3.1–3.9)	54.75 ± 21.15 (33.6–75.9)	6.8 ± 2.4 (4.4–9.2)	10.55 ± 1.45 (9.1–12)
Type II	40	27,579 ± 18,567.4 (9350–68,200)	82 ± 9.22 (57.5–92.2)	1.49 ± 1.29 (0.1–5.3)	148.98 ± 123.56 (42.8–427)	12.11 ± 3.62 (6.94–18.79)	6.95 ± 1.85 (3.7–9.2)
Type III	2	58,483 ± 6444 (52,039–64,927)	88.3 ± 3.8 (84.5–92.1)	4.15 ± 1.25 (2.9–5.4)	118 ± 26 (92–144)	13.11 ± 0.89 (12.22–14.01)	6.9 ± 0.9 (6–7.8)
Type IV	36	665.05 ± 629.14 (145–2510)	37.17 ± 13.48 (11–61.3)	1.69 ± 1.35 (0.1–5.5)	9.77 ± 11.93 (1–33.1)	7.55 ± 2.77 (4.79–13.18)	5.71 ± 1.94 (3.2–9.1)
Type V	8	3690 ± 3280.40 (650–8850)	44.87 ± 12.32 (34.5–65.9)	1.05 ± 0.79 (0.1–2.1)	5.3 ± 1.42 (4.9–7.2)	8.08 ± 2.21 (4.95–9.48)	6.11 ± 1.37 (3.9–7.7)
Type VI	1	27,980	88.3	3.5	99.4	9.4	5.5
Type VII	6	6286.66 ± 3669.94 (1430–10,300)	57.23 ± 2.84 (53.4–60.2)	1.33 ± 0.57 (0.4–2.1)	6.26 ± 4.98 (1.1–13)	7.29 ± 1.13 (5.7–8.17)	5.45 ± 1.08 (3.7–7.1)
Type VIII	12	12,956.67 ± 6641.03 (1040–20,900)	67.5 ± 8.11 (8.8–80.9)	13.06 ± 22.29 (2.7–62.9)	19.8 ± 7.49 (12.2–30)	9.13 ± 2.05 (6.25–10.89)	5.7 ± 1.22 (3.3–8.1)
Type IX	10	39,706.4 ± 23,995.85 (9200–73,400)	79.12 ± 11.18 (62–91.5)	8.0 ± 6.63 (2–16.7)	171.62 ± 60.95 (77.5–228)	15.26 ± 3.58 (10.2–19.91)	6.47 ± 1.38 (4.1–8.1)

**Table 2:** Distribution of aspirates with pathogen detection among the different LMNE types.

	Type II	Type III	Type VI	Type IX	Total
*Staphylococcus aureus*	14	1	0	4	19
*Cutibacterium acnes*	5	0	1	0	6
*Streptococcus dysgalactiae*	3	0	0	1	4
*Escherichia coli*	2	0	0	1	3
*Finegoldia magna*	2	0	0	0	2
*Staphylococcus epidermidis*	1	0	0	1	2
*Streptococcus pneumoniae*	1	0	0	0	1
*Staphylococcus warneri*	0	1	0	0	1
Total	28	2	1	7	38

**Table 3:** Distribution of the 51 septic arthritides across the different LMNE matrices and joints above and below the cut-off of the cell count of 50,000 cells /μL.

	Total	Cell Count >50,000/μL	Cell Count <50,000/μL
Type II	38	6	32
Type III	2	2	0
Type VI	1	0	1
Type IX	10	4	6
Total	51	12	39

**Table 4:** Overview of the different LMNE types with their graphical features, typical diagnosis and the required confirmatory tests.

LMNE Type	Graphical Features of LMNE Matrix	Typical Diagnosis	Required Confirmatory Tests
I (particle type)	Clusters in the noise field No peak in the erythrocyte curve	Crystal arthropathy	Microscopy, Histology, Uric acid
II (infection type)	Clusters in the field of neutrophil granulocytes No peak in the erythrocyte curve	Septic arthritis	Synovial cell count, %PMN, Microbial culture, CRP, Leukocytes
III (particle + infection type)	Clusters in the field of neutrophil granulocytes and in the noise field No peak in the erythrocyte curve	Combination of septic arthritis and crystal arthropathy	Microscopy, Histology, Synovial cell count, %PMN, Microbial culture, CRP, Leukocytes, Uric acid
IV (indifference type)	Unclear distribution pattern of clusters No peak in the erythrocyte curve	Reactive joint effusion	Clinical examination
V (haematoma type)	Clusters in the field of lymphocytes and neutrophils + Peak in the erythrocyte curve	Haemarthrosis	Clinical examination, Patient history
VI (haematoma + infection type)	Clusters in the field of neutrophils + Peak in the erythrocyte curve	Infected haemarthrosis	Synovial cell count, %PMN, Microbial culture, CRP, Leukocytes, Clinical examination, Patient history
VII (haematoma + particle type)	Clusters in the noise field and in the field of lymphocytes and neutrophils + Peak in the erythrocyte curve	Combination of haemarthrosis and crystal arthropathy or osteosynthetic caused debris	Clinical examination, Patient history, Uric acid, Microscopy, Histology
VIII (rheumatoid type)	Clusters in the field of neutrophils, lymphocytes, monocytes and large cells (on the far right)	Rheumatoid arthritis	Microscopy, Histology, Immature cells, CRP, Leukocytes, Rheumatic blood tests
IX (rheumatoid + infection type)	Clusters in the field of neutrophils and large cells (on the far right)	Combination of rheumatoid and septic arthritis	Microscopy, Histology, Rheumatic blood tests, Synovial cell count, %PMN, Microbial culture, CRP, Leukocytes

**Table 5:** Parameters for the different clinical diagnosis septic arthritis, crystal arthropathy, rheumatoid arthritis, haematoma and reactive effusion (mean ± standard deviation; minimum–maximum). In the four groups mentioned last, cases with concomitant infections were excluded.

	N	Cell Count (/μL)	% PMN	% LIMC	CRP (mg/L)	Leukocytes (10^3^/μL)	Uric Acid (mg/dL)
Septic Arthritis	51	31,162.82 ± 20,855.45 (9200–73,400)	81.89 ± 9.75 (57.5–92.9)	2.89 ± 4.13 (0.1–16.7)	149.76 ± 109.52 (39–427)	12.86 ± 3.67 (6.94–19.91)	6.78 ± 1.82 (3.7–9.2)
Crystal Arthropathy	2	82,128 ± 4635 (77,493–86,763)	26.2 ± 2.7 (23.5–28.9)	3.5 ± 0.4 (3.1–3.9)	54.75 ± 21.15 (33.6–75.9)	6.8 ± 2.4 (4.4–9.2)	10.55 ± 1.45 (9.1–12)
Rheumatoid Arthritis	16	10,096.25 ± 7953.18 (520–20,900)	58.91 ± 16.58 (8.8–80.9)	12.47 ± 20.69 (1.7–62.9)	21.1 ± 8.9 (12.2–308.9)	9.13 ± 2.05 (6.25–10.89)	5.81 ± 1.90 (2.1–8.1)
Haematoma	14	4802.85 ± 3684.1 (650–10,300)	50.17 ± 11.29 (34.5–65.9)	1.35 ± 0.65 (0.3–2.5)	5.78 ± 3.69 (1.1–13)	7.68 ± 1.80 (5.7–9.81)	5.05 ± 1.28 (3.5–7.5)
Reactive Effusion	34	2491.23 ± 7743.07 (145–33,410)	38.59 ± 16.98 (11–79.9)	2.0 ± 0.97 (0.3–3.7)	11.18 ± 10.55 (1–33.1)	7.92 ± 2.57 (5.15–13.18)	5.23 ± 1.33 (3.4–7.9)

**Table 6:** 2 × 2 contingency table evaluating the diagnostic performance of the LMNE matrix.

LMNE Type	Septic per Definition	Aseptic per Definition	Total
Infectious (Type II, III, VI, IX)	51	4	55
Non-infectious (Type I, IV, VII, VIII)	0	62	62
Total	51	66	117

## Data Availability

The data supporting the findings of this study are not publicly available due to ethical and privacy restrictions, but are available from the corresponding author upon reasonable request.

## Abbreviations

The following abbreviations are used in this manuscript:

CRP	C-reactive protein
LIMC	Large immature cells
LMNE	Leukocytes-Monocytes-Neutrophils-Eosinophils
PMN	Polymorphonucleated neutrophils
